# Clinical Factors Associated with Binge-Eating Episodes or Purging Behaviors in Patients Affected by Eating Disorders: A Cross-Sectional Study

**DOI:** 10.3390/jpm14060609

**Published:** 2024-06-07

**Authors:** Alice Caldiroli, Letizia Maria Affaticati, Sara Coloccini, Francesca Manzo, Alberto Scalia, Enrico Capuzzi, Davide La Tegola, Fabrizia Colmegna, Antonios Dakanalis, Maria Salvina Signorelli, Massimiliano Buoli, Massimo Clerici

**Affiliations:** 1 Department of Mental Health, Fondazione IRCCS San Gerardo dei Tintori, via G.B. Pergolesi 33, 20900 Monza, Italy; e.capuzzi1@campus.unimib.it (E.C.); davide.lategola@irccs-sangerardo.it (D.L.T.); fabrizia.colmegna@irccs-sangerardo.it (F.C.); antonios.dakanalis@unimib.it (A.D.); massimo.clerici@unimib.it (M.C.); 2 Department of Medicine and Surgery, University of Milan Bicocca, via Cadore 38, 20900 Monza, Italy; letizia.affaticati@gmail.com (L.M.A.); f.manzo5@campus.unimib.it (F.M.); a.scalia5@campus.unimib.it (A.S.); 3 Department of Clinical and Experimental Medicine, Institute of Psychiatry, University of Catania, 95123 Catania, Italy; sara.coloccini@gmail.com (S.C.); maria.signorelli@unict.it (M.S.S.); 4 Department of Neurosciences and Mental Health, Fondazione IRCCS Ca’Granda Ospedale Maggiore Policlinico, 20122 Milan, Italy; massimiliano.buoli@unimi.it; 5 Department of Pathophysiology and Transplantation, University of Milan, 20122 Milan, Italy

**Keywords:** clinical markers, eating disorders, objective binge-eating episodes, purging, severity of illness

## Abstract

The aim of the present study was to investigate the potential associations between clinical/socio-demographic variables and the presence of purging/binge-eating episodes in eating disorders (EDs). Clinical/socio-demographic variables and psychometric scores were collected. Groups of patients were identified according to the presence or absence of purging or objective binge-eating episodes (OBEs) and compared through *t*-test and chi-square tests. Binary logistic regression analyses were run. A sample of 51 ED outpatients was recruited. Patients with purging behaviors had a longer duration of untreated illness (DUI) (t = 1.672; *p* = 0.019) and smoked a higher number of cigarettes/day (t = 1.061; *p* = 0.030) compared to their counterparts. A lower BMI was associated with purging (OR = 0.881; *p* = 0.035), and an older age at onset showed a trend towards statistical significance (OR = 1.153; *p* = 0.061). Patients with OBEs, compared to their counterparts, were older (t = 0.095; *p* < 0.001), more frequently presented a diagnosis of bulimia or binge-eating disorder (χ^2^ = 26.693; *p* < 0.001), a longer duration of illness (t = 2.162; *p* = 0.019), a higher number of hospitalizations (t = 1.301; *p* = 0.012), and more often received a prescription for pharmacological treatment (χ^2^ = 7.864; OR = 6.000; *p* = 0.005). A longer duration of the last pharmacological treatment was associated with OBE (OR = 1.569; *p* = 0.046). In contrast to purging, OBE was associated with a more complicated and severe presentation of ED. A lower BMI and a later age at onset, as well as long-lasting previous pharmacological treatments, may predict the presence of purging/binging. Further research is needed to thoroughly characterize ED features and corroborate our preliminary findings.

## 1. Introduction

Eating disorders (EDs) are mental health conditions characterized by dysfunctional eating habits associated with concerns about weight and shape [1]. EDs represent a public health problem of growing importance due to their prevalence, early onset, and complex multifactorial etiology. EDs are estimated to affect 1.69% of the global population over the course of a lifetime [2]. Rates are particularly high for anorexia nervosa (AN), bulimia nervosa (BN), and binge eating disorder (BED), with a well-documented lifetime prevalence of 0.16%, 0.63%, and 1.53%, respectively [2]. Otherwise specified feeding or eating disorder (OSFED) is a new and somewhat less studied diagnostic entity, with extant reports indicating a 3-month prevalence of 3.2% [2]. In more than 20% of affected patients, EDs exhibit a chronic course [3], leading to both psychiatric and medical complications. Thus, early diagnosis and treatment initiation are essential to prevent chronicity and curtail public health burdens.

According to the Fifth edition of the Diagnostic and Statistical Manual of Mental Disorders (DSM-5) [1], AN is defined as a restriction of energy intake leading to low body weight, in association with an intense fear of weight gain and disturbances in the perception of one’s body weight or shape. AN is further subdivided into restricting type and binge-eating/purging type, depending on the primary methods employed to accomplish weight loss [1]. BN is characterized by recurrent binge-eating episodes and inappropriate compensatory behaviors; self-evaluation of affected individuals is heavily dependent upon one’s body shape and/or weight [1]. BED is defined by recurrent binge-eating episodes causing significant distress without associated compensatory behaviors [1]. OSFED refers to presentations in which symptoms characteristic of an ED that cause significant distress or impairment do not meet the full criteria for any ED diagnostic class (e.g., atypical AN, BN of low frequency/limited duration, BED of low frequency/limited duration, purging disorder) [1].

As per the DSM-5 [1], the level of AN severity is based on the current body mass index (BMI), whilst the severity of BN and BED is determined by the number of binge-eating episodes per week. While BMI and the number of weekly binge-eating episodes are useful parameters for initial severity assessment [4], they do not fully capture the complexity of ED presentations and clinical courses. Several additional instruments are commonly employed in clinical practice to gain a more comprehensive understanding of EDs’ presentation and progression. Among these, the Eating Disorder Inventory (EDI) [5,6] and the Eating Disorder Examination (EDE) are prominent [7]. The EDI-3 is a standardized, self-report questionnaire assessing the main symptoms and psychological features of EDs [8]. The EDE-17 is a semi-structured, clinician-administered interview designed to evaluate specific aspects of ED psychopathology, as well as the severity of dysfunctional eating behaviors and attitudes [9].

Dysfunctional eating behaviors are core aspects of eating pathology and are present across a wide range of ED diagnoses [1]. They can be broadly categorized as compensatory behaviors and overeating behaviors. Compensatory behaviors are inappropriate weight-control strategies and may be further divided into purging (e.g., self-induced vomiting and use of laxatives, diuretics, and enemas) and non-purging behaviors (e.g., compulsive exercise, fasting, and use of diet pills). Overeating behaviors include objective binge-eating episodes (OBEs) and subjective binge-eating episodes (SBEs) [9]. OBEs are episodes of binge-eating as defined by the DSM-5 [1]—i.e., characterized by the sensation of loss of control over eating in a discrete time frame, during which the amount of food consumed is ‘definitely larger than what most people would eat’ under similar circumstances. An SBE is a form of overeating associated with a loss of control and perceived by the individual as a binge episode but which involves the consumption of a small or moderate amount of food [10]. While some studies have shown the utility of SBEs as clinical parameters/severity indicators [11,12], there is still international dispute around SBEs’ recognition and formal inclusion in the DSM diagnostic criteria [13].

Binge-eating and purging behaviors are particularly worrisome due to their association with serious physical complications. Purging behaviors have been linked to gastrointestinal sequelae, as well as electrolyte imbalances and cardiac arrhythmias [14]. Binge-eating has been associated with an increased risk of metabolic syndrome and its components—particularly hypertriglyceridemia, hypertension, and high fasting glucose/type 2 diabetes [15]. The literature also indicates a positive relation between binge–purge behaviors and menstrual abnormalities, including amenorrhea and irregular menses—especially among individuals who smoke [16,17].

Along with medical complications, the presence of binging and/or purging has been associated with a more complex and severe ED course, including greater treatment resistance and risk of life-threatening malnutrition [18,19]. Moreover, patients who engage in these behaviors tend to report more ED symptoms, along with higher levels of anxiety, low affect, and self-harm [20,21,22]. Psychiatric comorbidities among patients who binge and/or purge are frequent, particularly with substance abuse [23] and cluster B personality disorders (PDs) [24,25]. Finally, the frequency of binge eating has been associated with greater levels of functional impairment in personal, social, and cognitive domains [26].

The above findings suggest that binging and purging are predictors of adverse outcomes and of a more complicated disease course per se, regardless of the primary diagnosis. Most studies to date have investigated these behaviors in relation to a specific ED diagnosis (e.g., binge/purge vs. restrictive AN) [25,26] and a specific frequency threshold (e.g., one/two purging episodes per week) [27,28]. On the other hand, there is scant literature focusing on the impact of binging and purging from a trans-diagnostic perspective, regardless of their frequency, which could offer valuable insights for personalized medicine. Among the few available transdiagnostic studies, O’Kearney et al. [14] and Dalle Grave et al. [29] compared ED patients with and without purging behaviors; both found significantly higher levels of depression in purgers compared to non-purgers. The same authors also found higher scores on several items and subscales of major questionnaires for EDs (e.g., more severe weight and shape concern, higher levels of disordered eating) in the purging group [14,29]. Our literature review did not identify any studies analyzing binge-eating behaviors across a sample of patients with different primary diagnoses of EDs. In addition, to our knowledge, no study has yet investigated both behaviors in the same patient population. The present study sought to investigate the individual associations of binging and purging with clinical and socio-demographic variables relevant to the course and outcomes of EDs in order to shed light on their potential correlates. We hypothesized that the presence of binging or purging would negatively affect the clinical course of EDs, independently of ED diagnosis. A better understanding of how binging and purging behaviors affect the course of illness can potentially inform patients’ risk stratification and offer insights into the lack of treatment response. In addition, it may guide the development of personalized treatments more specifically aimed at reducing these behaviors, regardless of the primary ED diagnosis. Given the high prevalence, chronicity, and public health burden of these diseases, investing in personalized assessments and treatment strategies appears essential.

## 2. Materials and Methods

### 2.1. Study Design and Participants

This is a cross-sectional monocentric study. The research project was reviewed and approved by the local Ethical Committee (Comitato Etico Territoriale—CET3) with protocol number 3951. The study was conducted according to the provisions of the latest version of the Declaration of Helsinki.

Outpatients presenting to our specialist ED clinic (Fondazione IRCCS San Gerardo dei Tintori, Monza, Italy) from 23 August 2022 to 30 April 2023 were consecutively recruited for the present study. Inclusion criteria were: (1) age 17–60 years; (2) fluency in Italian language; (3) ability and willingness to sign the written informed consent; (4) diagnosis of AN, BN, BED, or OSFED according to the DSM-5 criteria [1]. Exclusion criteria were: (1) pregnancy or breastfeeding; (2) intellectual disability; (3) presence of a severe organic disease that could affect eating attitudes and weight (e.g., neoplasia-induced cachexia, endocrine imbalance).

### 2.2. Assessment

ED diagnostic evaluations were conducted by an expert senior psychiatrist in accordance with DSM-5 criteria during the patients’ first psychiatric visit. Diagnoses were confirmed by administering the Italian version of the Eating Disorder Examination-EDE-Interview-17.0 [7,9]. The EDE subscale scores (Restraint, Eating Concern, Shape Concern, and Weight Concern) were calculated, and the EDE global score was derived as the mean of the four subscale scores [9]. The presence and frequency of the following purging and binge-eating behaviors in the last month were also determined using the EDE: self-induced vomiting, laxative misuse, diuretic misuse, compulsive exercise, objective and subjective binge-eating episodes, and objective overeating.

In addition to the above, the following socio-demographic and clinical data were collected from the patients themselves or from their relatives during the patients’ first psychiatric visit: age, gender, education (years), work, marital status, smoke, number of cigarettes/day, dietary habits, age at onset, diagnosis, duration of illness (months), duration of untreated illness (DUI), BMI, presence and type of family history of mental disorders, obstetrical complications, presence and type of lifetime substance use disorders, poly-substance use disorders, presence and type of psychiatric comorbidity, multiple psychiatric comorbidity, lifetime psychotic symptoms, presence and type of comorbid personality disorder, presence of lifetime suicide attempts, presence and number of lifetime hospitalizations, presence and type of main current pharmacological treatment, current poly-pharmacotherapy therapy and psychotherapy, pharmacological treatment prescription, type of pharmacotherapy prescription, duration of the last pharmacological treatment (months), medical comorbidity, multiple medical comorbidity, comorbidity with thyroid disorders, hypercholesterolemia, diabetes, amenorrhea. DUI was defined as the time elapsing between ED onset and initiation of the first appropriate treatment strategy, according to international guidelines [30,31,32]. BMI was calculated by dividing the weight in kg by the height in m^2^.

Additionally, the severity of anxiety and depressive symptoms, as well as the global clinical severity, were measured using the following psychometric scales: Clinical Global Impression (CGI) [33] severity subscale, Hamilton Anxiety Rating Scale (HAM-A) [34], Hamilton Depression Rating Scale (HAM-D) [35], Montgomery and Åsberg Depression Rating Scale (MADRS) [36]. The Italian versions of these psychometric scales, along with their psychometric properties, are reported in the book by Conti (1999) [37].

### 2.3. Statistical Analyses

Descriptive analyses were conducted on the entire sample. Frequencies were calculated as percentages for qualitative variables and as means ± standard deviation for quantitative variables.

For the purpose of our analyses, the sample was divided into two groups of patients based on the presence or absence of purging behaviors/compulsive exercise. These two groups were compared in terms of qualitative and continuous variables using chi-square tests and Student’s *t*-tests, respectively. The Benjamini–Hochberg test for False Discovery Rate was applied to correct for multiple comparisons. Binary logistic regression analysis was then conducted; continuous variables that were statistically significant (*p* ≤ 0.05) in Student’s *t*-tests served as independent variables, while the presence of purging episodes served as the dependent variable. 

The sample was then divided into two groups based on the presence or absence of OBEs. These were compared in terms of qualitative and continuous variables using chi-square tests and Student’s *t*-tests, respectively. The Benjamini–Hochberg test for False Discovery Rate was applied to correct for multiple comparisons. Binary logistic regression analysis was then run; continuous variables that were statistically significant (*p* ≤ 0.05) in Student’s *t*-tests served as independent variables, with the presence of OBEs serving as the dependent variable. Two variables (number of self-induced vomiting episodes and of compulsive exercise in the last month) that were found to be statistically significant in the Student’s *t*-tests were excluded from the regression model due to a high risk of collinearity.

The goodness of fit for both models was assessed using Hosmer–Lemeshow [38] and the Omnibus tests.

Statistical analyses were performed using The Statistical Package for Social Sciences (SPSS) for Windows (version 28.0). The level of statistical significance was set at *p* ≤ 0.05.

**Figure 1:**
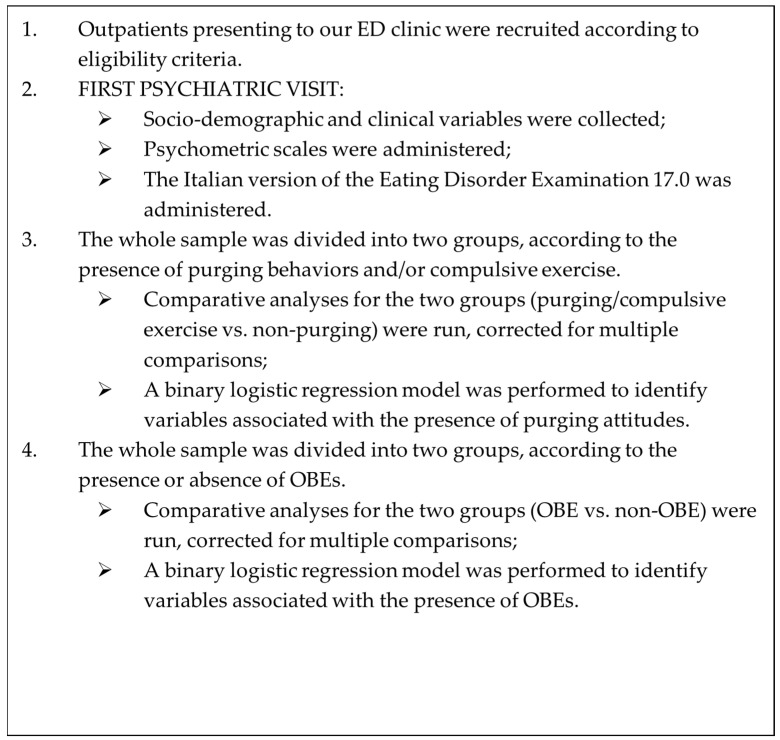
Methodological steps of the present study.

See Figure 1 for a summary of methodological steps of the study.

## 3. Results

### 3.1. Descriptive Analyses

A total of 51 ED outpatients were recruited. The mean age of the entire sample was 24.8 (±8.19) years. Twenty-six patients (51.0%) reported purging behaviors, and nineteen (37.3%) reported OBEs in the last month. Descriptive analyses conducted on the whole sample and on subgroups are reported in Table 1.

### 3.2. Group Comparisons According to the Presence/Absence of Purging Episodes

As can be expected, based on the results of the chi-square and Students’ *t*-tests, patients in the purging group more frequently presented the following compensatory behaviors: self-induced vomiting (χ^2^ = 20.433; *p* < 0.001; OR = 2.364–95% confidence interval (CI): 1.509–3.703), laxatives misuse (χ^2^ = 10.508; *p* = 0.001; OR = 1.529–95% CI: 1.156–2.023), and compulsive exercise (χ^2^ = 16.776; *p* < 0.001; OR = 2.000–95% CI: 1.362–2.937). They also reported a higher number of self-induced vomiting acts (t = 2.380; *p* < 0.001), laxatives use (t = 2.014; *p* < 0.001), diuretics use (t = 1.414; *p* = 0.003), and compulsive exercise (t = 4.084; *p* < 0.001).

Moreover, according to the Student’s *t*-tests, patients presenting purging behaviors or compulsive exercise had an older age at onset (t = 2.721; *p* = 0.015), a longer DUI (t = 1.672; *p* = 0.019), a lower BMI (t = 2.173; *p* < 0.001), and smoked a higher number of cigarettes/day (t = 1.061; *p* = 0.030) compared to their counterparts. 

Complete results for these comparisons are reported in Table 2.

In regression analysis, a lower BMI was associated with the presence of purging behaviors/compulsive exercise (OR = 0.881; *p* = 0.035), and a later age at onset showed a trend towards statistical significance (OR = 1.153; *p* = 0.061) (Table 3).

The goodness-of-fit test (Hosmer and Lemeshow Test: χ^2^ = 2.844; df = 7; *p* = 0.899) showed that the model, including age at onset, number of cigarettes/day, BMI, and DUI, as possible predictors of the presence of purging behaviors, was reliable, allowing for a correct classification of 74.5% of the cases. In addition, the model was overall significant (Omnibus test: χ^2^ = 16.113; df = 4; *p* = 0.003).

### 3.3. Group Comparisons According to the Presence/Absence of OBEs

According to the chi-square tests, patients in the OBE group more frequently presented a diagnosis of BN or BED (χ^2^ = 26.693; *p* < 0.001) and more often received a pharmacological treatment prescription (χ^2^ = 7.864; *p* = 0.005; OR = 6.000–95% CI: 1.616–22.283).

As can be expected, based on the results of the Student’s *t*-tests, patients with OBEs reported a higher number of OBEs (t = 5.895; *p* < 0.001), SBEs (t = 1.296; *p* = 0.012), and objective overeating episodes (t = 1.162; *p* = 0.026). They also presented a higher number of episodes of self-induced vomiting (t = 2.675; *p* < 0.001) and compulsive exercise (t = 1.212; *p* = 0.004).

According to the Student’s *t*-tests, patients in the OBE group were also older (t = 0.095; *p* < 0.001), had a longer duration of illness (t = 2.162; *p* = 0.019), and reported a higher number of hospitalizations (t = 1.301; *p* = 0.012) and a longer duration of the last pharmacological treatment (t = 3.189; *p* < 0.001). 

Complete results for these comparisons are reported in Table 2.

In regression analysis, a longer duration of the last pharmacological treatment was associated with the presence of OBEs (OR = 1.569; *p* = 0.046) (Table 4).

The goodness-of-fit test (Hosmer and Lemeshow Test: χ^2^ = 5.749; df = 8; *p* = 0.675) showed that the model including age, duration of illness, duration of the last pharmacological treatment, and number of hospitalizations as possible predictors of the presence of OBEs was reliable, correctly classifying 78.6% of the cases. The model was overall significant (Omnibus test: χ^2^ = 11.652; df = 4; *p* = 0.020). 

## 4. Discussion

The present study sought to explore clinical and socio-demographic variables associated with purging and binging behaviors among a sample of outpatients with different ED diagnoses. In the final model, the presence of purging episodes/compulsive exercise was associated with a lower BMI and a later age at onset (the latter with a trend towards statistical significance). In addition, purging/compulsive exercise was associated with a longer DUI and a greater number of cigarettes smoked daily in Student’s *t*-tests, but these associations did not survive regression analyses. On the other hand, patients with OBEs more frequently presented a BN or BED diagnosis, a pharmacotherapy prescription at first visit, and a longer duration of the last pharmacological treatment. Student’s *t*-tests also revealed older age, longer duration of illness, a high number of previous hospitalizations, and more frequent episodes of self-induced vomiting and compulsive exercise in the OBE group. However, these associations were not confirmed in regression analyses. 

It is worth noting that most patients in our sample had mild to moderate depressive scores, which were lower compared to several ED samples in the literature [14,39]. This difference may be due to a variety of factors. For instance, we conducted our study on a sample of adult outpatients, while oftentimes studies have been conducted on adolescent samples [39] or, less commonly, on inpatients [29]. In addition, we measured depression using the HAM-D, whilst other studies (e.g., [14]) used self-report instruments, which may have a greater tendency to amplify symptomatology.

The prevalence of purging behaviors in our sample was 51.0%, in line with a previous study comparing purging across patients with different ED diagnoses (56.6%) [29]—although higher frequencies among ED patients have also been reported [14]. This percentage is noteworthy considering the medical complications associated with purging, which may be severe and potentially lethal [40].

A greater premorbid BMI has been repeatedly recognized as a risk factor for developing purging attitudes in both previously healthy populations and ED patients [41,42]. Prior studies have also reported a higher current and prospective BMI in purgers compared to non-purgers [29,43], indicating that a higher weight might sustain the persistence of purging behaviors in addition to facilitating their initiation. These observations appear in contrast with our findings of a lower BMI among patients who purge. However, the high prevalence of AN among purgers in our sample might partly account for this difference. In addition, one of the above studies found that BMI was no longer significantly associated with purging after adjusting for weight dissatisfaction [43], suggesting that perceived rather than actual body shape may be the principal drive towards purging methods.

In terms of age at onset, our findings align with prior research reporting older age at onset in patients with binge-eating/purging AN (AN-BP) as compared with those with restricting AN (AN-R) [44]. Moreover, there is evidence suggesting a later onset of purging relative to other pathological eating habits, including binging, in previously healthy adolescents [45]. Interestingly, dieting seems to be the first symptom of AN, with purging and other attitudes occurring later in the progression of the disorder [46]. Furthermore, the transition from AN-R to binge–purge behaviors/disorders during follow-up is well documented in the literature [42], while there is no substantial evidence of the reverse crossover. Taken together, these studies suggest that purging tends to occur later compared to other ED behaviors, with implications in terms of preventive measures.

While the association between binge–purge behaviors and substance use is well-documented [23], fewer studies have investigated the independent relationship between purging behaviors and the consumption of addictive substances. A multi-population study on adolescent girls [21] found purging to be significantly associated with smoking, binge drinking, and drug use, even after adjusting for the presence of binge-eating behavior. In our sample, patients who purged did not abuse illicit drugs or alcohol but did have a higher daily cigarette consumption. The lack of an association with drugs and alcohol use might be due to several factors, including differences in the definition of substance use (e.g., we did not record casual drug use), as well as the older mean age of our sample.

Regarding binge-eating behaviors, the first notable result was that 40% of the total sample presented OBEs. Recurrent binge-eating episodes are highly distressing, as indicated in the DSM-5 [1], and have risen among the general population over the past 18 years [47]. In particular, diagnoses of BN and BED appear to have doubled in the last twenty years [48]. Consistently with these findings, BN and BED collectively accounted for 37.3% of ED diagnoses in our sample.

As a second finding, univariate analyses indicated that subjects with OBEs more frequently presented a diagnosis of BN or BED. Indeed, OBEs represent the core features of BN and BED according to the DSM-5 [1], although binge-eating behaviors may also occur in patients with AN and OSFED. However, in our sample, anorexic patients did not report such behaviors. Interestingly, a recent meta-analysis of 20 original articles found that OBEs are more prevalent in typical AN compared to atypical AN [49] and, overall, supported the hypothesis that these two AN subtypes do not substantially differ in terms of severity and need for care. We did not directly compare AN and atypical AN, as the latter was included in the OSFED diagnostic category. As expected, patients with OBEs also reported significantly more episodes of self-induced vomiting and compulsive exercise compared to those without OBE. This may be due to the slightly higher number of BN patients compared to BED patients in our sample, although to date, the association between binge eating and compensatory behaviors is still debated [50].

Thirdly, our analyses showed that patients with OBE had a longer duration of illness with respect to their counterparts. This finding may be related to the older age of individuals with OBE compared to patients without binge eating in our sample. Naturally, age and duration of illness are intrinsically correlated. Nevertheless, it has been reported that 29% of subjects with OBEs exhibit a chronic ED course [51]. Of note, a longer duration of illness has been linked to nonresponse to treatment and poor response to previous treatments in EDs [52] and appears to mediate the relationship between severity and functional impairment in EDs [53].

As a fourth observation, patients with OBEs were more frequently prescribed pharmacotherapy at their first psychiatric visit. In addition, a longer duration of the last pharmacological treatment was associated with the presence of OBEs in our study. From a diagnostic standpoint, a longer treatment duration has been associated with lower mortality rates in AN and higher recovery rates in BN [51]. Based on these findings, we can speculate that treating ED patients for a longer time may lead to a better prognosis. However, our results referred to the presence of OBEs independently of diagnosis and solely to pharmacological treatment (i.e., we did not consider psychotherapy). Of note, some authors have shown a decrease in binge-eating episodes following psychological or behavioral treatments [54] and that pharmacotherapy alone or in combination was less effective than psychotherapy in addressing binge-eating and depressive symptoms, even in the long term [55,56]. Moreover, the most commonly prescribed medications in our sample were zolpidem, sertraline, or fluoxetine, indicating that pharmacological treatment in patients reporting OBE may primarily target concomitant symptoms, such as insomnia or depression. Nonetheless, the stability of treatment effects and long-term efficacy over 12 months remain unclear for most compounds, necessitating further research [31,57].

Our results support the hypothesis that OBEs may be more resistant in responding to pharmacotherapy and may require a longer treatment duration. This is further supported by our finding of a higher number of hospitalizations in patients with OBEs, which provides further evidence of a poorer treatment response in patients with binge-eating behaviors. Of note, a meta-analysis showed that different clinical settings similarly influence the weight of patients affected by BN or BED [58].

Finally, in our univariate analyses, the presence of poly-substance misuse, as well as comorbid anxiety disorders and borderline PD, was found to be more common in patients with OBEs, regardless of the specific eating disorder diagnosis (although these results did not remain significant after corrections for multiple comparisons). Extensive evidence in the literature supports the association between EDs and substance use disorders (SUDs) [23], even among adolescents and college students [59,60]. Particularly, the connection between binge eating and SUDs has been extensively studied and demonstrated in various countries [61,62,63], suggesting that these two disorders may share underlying neurobiological mechanisms regardless of food availability. Importantly, the impact of SUDs on mortality rates in EDs appears to be additive compared to control subjects without SUDs [64], and the comorbidity between EDs and SUDs increases the risk for somatic diseases beyond the effects of each disorder independently [65]. It is noteworthy that drugs and food have different neurobiological effects, with drugs having a more pronounced impact than food on neurobiological processes [66]. 

Among comorbid symptoms, self-harm behaviors are strongly related to EDs [67]; however, we did not observe an increased presence/number of lifetime suicide attempts in patients with OBEs compared to their counterparts. This may be explained by the distinction between suicidal and non-suicidal self-harm [68], with the latter being more characteristic of borderline PD [69]. While conducting our research, we only collected data on suicide attempts, not self-injuries, which could explain the absence of such behaviors in our sample. Some authors have investigated predictors of suicide attempts in EDs and concluded that the diagnostic category is the most significant factor [70]. Given that our sample included a mix of ED diagnoses, it may not have been sufficiently representative of each individual diagnosis to detect self-harm behaviors. Moreover, borderline PD was found to be more represented in binge-eating patients in our sample, consistent with previous studies [25,71]. Of note, a comorbidity with a PD in patients suffering from BN represents a risk factor for increased all-cause mortality [72]. 

It is plausible that certain personality traits sustain specific pathological processes seen in both SUD and BED, thus supporting the hypothesis of shared etiological factors. Moreover, common clinical and psychopathological features, such as external cue reactivity, craving, emotion dysregulation, and impulsivity, along with alterations of the dopaminergic system, may favor chronicity in binge eating [61,66]. Nevertheless, the exact nature of the association between BED and PDs remains to be clarified [71].

### Strengths and Limitations

The main strength of the present study is the investigation of ED patterns from a trans-diagnostic perspective, which remains relatively less represented in the current literature. Within the context of precision medicine, a transdiagnostic approach holds the potential for uncovering meaningful associations that can inform personalized assessments and clinical interventions. In addition, the evaluation of ED psychopathology through a structured, investigator-based interview (EDE-17D) allows more reliable identification of complex behavioral features like OBE [73,74]. Nonetheless, certain limitations should also be considered. Firstly, our sample was relatively small and composed solely of outpatients. Thus, our results require validation from studies employing larger samples, preferably composed of both in- and outpatients. The employment of a control group would also lend greater strength to our findings. Moreover, due to our limited sample size, we were unable to further divide our participants into subgroups that have been previously identified as clinically relevant—e.g., patients with multiple purging behaviors [75]. A further limit of our study is the lack of analyses on quantitative measures of symptom severity in favor of a qualitative investigation of binging/purging symptoms. Finally, the use of other tools, such as the Temperament and Character Inventory [76], may offer greater precision in pinpointing significant personality traits compared to categorical diagnoses of PD.

## 5. Conclusions

Taken as a whole, our findings suggest that the presence of OBEs is associated with a more complex and severe presentation of EDs. This highlights the potential role of binge-eating episodes as markers of severity in EDs. The presence of OBEs warrants attention from clinicians, and increasing the screening rates of binge-eating behaviors might be crucial in promptly identifying patients at risk of developing a more severe ED [77]. Further studies are needed to explore the role of compensatory behaviors in EDs and their clinical implications in order to inform preventive measures and treatment strategies.

## Figures and Tables

**Table 1:** Descriptive analyses of the total sample and of groups defined by the presence/absence of objective binge-eating episodes or purging behaviors.

Variables	Total Sample *N* = 51		Objective Binge-Eating Episodes		Purging Behaviors	
			YES (*n* = 19)	NO (*n* = 32)	YES (*n* = 26)	NO (*n* = 25)
Age	24.8 (±8.19)		24.9 (±7.9)	24.7 (±8.5)	27.1 (±8.6)	22.4 (±7.1)
Gender	Male	3 (5.9%)	0	3 (9.4%)	1 (3.8%)	2 (8.0%)
	Female	48 (94.1%)	19 (100%)	29 (90.6%)	25 (96.2%)	23 (92.0%)
Education (years)	12.9 (±3.25)		13.0 (±3.6)	12.8 (±3.1)	13.4 (±2.9)	12.3 (±3.5)
Work	Unemployed	3 (5.9%)	2 (10.5%)	1 (3.1%)	0	3 (12.0%)
	Student	30 (58.8%)	10 (52.6%)	20 (62.5%)	13 (50.0%)	17 (68.0%)
	Employed	18 (35.3%)	7 (36.9%)	11 (34.4%)	13 (50.0%)	5 (20.0%)
Marital Status Missing *n* = 4	Single/separated	24 (51.1%)	9 (52.9%)	15 (50.0%)	12 (48.0%)	12 (54.5%)
	Married	23 (48.9%)	8 (47.1%)	15 (50.0%)	13 (52.0%)	10 (45.5%)
Smoke Missing *n* = 3	No	42 (87.5%)	16 (88.9%)	26 (86.7%)	22 (84.6%)	20 (91.9%)
	Yes	6 (12.5%)	2 (11.1%)	4 (24.3%)	4 (15.4%)	2 (9.1%)
Number of cigarettes/day Missing *n* = 3	1.6 (±4.85)		1.7 (±5.1)	1.6 (±4.8)	2.3 (±5.9)	0.8 (±3.2)
Dietary habits Missing *n* = 4	Omnivorous	44 (93.7%)	14 (82.3%)	30 (100%)	22 (88.0%)	22 (100%)
	Vegetarian	2 (4.3%)	2 (11.8%)	0	2 (8.0%)	0
	Hyperproteic	1 (2.0%)	1 (5.9%)	0	1 (4.0%)	0
Age at onset	19.7 (±6.06)		18.4 (±6.1)	20.4 (±6.0)	21.8 (±7.1)	17.4 (±3.6)
Diagnosis	AN	21 (41.1%)	0	21 (65.6%)	12 (46.2%)	9 (36.0%)
	BN	11 (21.6%)	9 (47.4%)	2 (6.3%)	9 (34.6%)	2 (8.0%)
	BED	8 (15.7%)	6 (31.6%)	2 (6.3%)	1 (3.8%)	7 (28.0%)
	OSFED	11 (21.6%)	4 (21.0%)	7 (21.8%)	4 (15.4%)	7 (28.0%)
Duration of illness (months)	58.4 (±65.05)		84.0 (±83.6)	44.0 (±47.5)	65.9 (±65.1)	50.3 (±65.4)
DUI (months)	29.8 (±44.94)		36.8 (±56.9)	25.8 (±37.1)	39.8 (±58.1)	18.9 (±19.9)
BMI	22.9 (±6.75)		27.6 (±6.2)	20.2 (±5.5)	20.7 (±4.3)	25.3 (±8.0)
Family history of mental disorders Missing *n* = 2	No	29 (56.9%)	9 (50%)	20 (60.6%)	15 (62.5%)	14 (56.0%)
	Yes	20 (39.1%)	9 (50.0%)	11 (39.4%)	9 (37.5%)	11 (44.0%)
Type of family history Missing *n* = 2	None	29 (59.2%)	9 (50.0%)	20 (64.5%)	15 (62.5%)	14 (56.0%)
	Major depression	8 (16.3%)	3 (16.7%)	5 (16.1%)	4 (16.7%)	4 (16.0%)
	Anxiety disorders	2 (4.1%)	2 (11.1%)	0	2 (8.3%)	0
	SUD	1 (2.0%)	1 (5.5%)	0	0	1 (4.0%)
	EDs	9 (18.4%)	3 (16.7%)	6 (19.4%)	3 (12.5%)	6 (24.0%)
Obstetrical complications	None	47 (92.1%)	16 (84.1%)	31 (96.9%)	23 (88.5%)	24 (96.0%)
	Low weight (<2500 g)	2 (3.9%)	1 (5.3%)	1 (3.1%)	2 (7.7%)	0
	Premature (<32 weeks)	1 (2.0%)	1 (5.3%)	0	1 (3.8%)	0
	Cord hypoxia	1 (2.0%)	1 (5.3%)	0	0	1 (4.0%)
Lifetime substance use disorders Missing *n* = 1	No	45 (90.0%)	15 (83.3%)	30 (93.7%)	22 (84.6%)	23 (95.8%)
	Yes	5 (10.0%)	3 (6.7%)	2 (6.3%)	4 (5.4%)	1 (4.2%)
Main type of substance use disorder Missing *n* = 1	None	45 (90.0%)	15 (83.3%)	30 (93.7%)	22 (84.6%)	23 (95.8%)
	Cannabinoids	2 (4.0%)	0	2 (6.3%)	2 (7.8%)	0
	Opioids	1 (2.0%)	1 (5.4%)	0	0	1 (4.2%)
	Cocaine	1 (2.0%)	1 (5.4%)	0	1 (3.8%)	0
	Alcohol	1 (2.0%)	1 (5.4%)	0	1 (3.8%)	0
Poly-substance use disorders Missing *n* = 1	No	48 (96.0%)	16 (88.9%)	32 (100%)	25 (96.2%)	23 (95.8%)
	Yes	2 (4.0%)	2 (11.1%)	0	1 (3.8%)	1 (4.2%)
Psychiatric comorbidity Missing *n* = 2	No	36 (73.5%)	9 (52.9%)	27 (84.3%)	17 (68.0%)	19 (79.2%)
	Yes	13 (26.5%)	8 (47.1%)	5 (15.7%)	8 (32.0%)	5 (20.8%)
Type of psychiatric comorbidity Missing *n* = 2	None	36 (73.5%)	9 (52.9%)	27 (84.4%)	17 (70.8%)	19 (79.2%)
	Major depression	7 (14.3%)	3 (17.7%)	4 (12.5%)	3 (12.5%)	4 (16.6%)
	Anxiety disorders	5 (10.2%)	5 (29.4%)	0	4 (16.6%)	1 (4.2%)
	OCD	1 (2.0%)	0	1 (3.1%)	1 (4.1%)	0
Multiple psychiatric comorbidity Missing *n* = 2	No	49 (100%)	17 (100%)	32 (100%)	25 (100%)	24 (100%)
Lifetime psychotic symptoms Missing *n* = 1	No	50 (100%)	18 (100%)	32 (100%)	26 (100%)	24 (100%)
Personality disorder Missing *n* = 6	No	34 (75.6%)	8 (53.3%)	26 (86.7%)	17 (70.8%)	17 (80.9%)
	Yes	11 (24.4%)	7 (46.7%)	4 (13.3%)	7 (29.2%)	4 (19.1%)
Type of personality disorder Missing *n* = 6	None	34 (75.6%)	8 (53.3%)	26 (86.7%)	17 (70.8%)	17 (81.0%)
	Schizotypic	1 (2.2%)	0	1 (3.3%)	1 (4.2%)	0
	Borderline	7 (15.6%)	5 (3.3%)	2 (6.7%)	5 (20.8%)	2 (9.5%)
	Hystrionic	2 (4.4%)	2 (3.4%)	0	0	2 (9.5%)
	Obsessive-compulsive	1 (2.2%)	0	1 (3.3%)	1 (4.2%)	0
Lifetime suicide attempts	No	51 (100%)	19 (100%)	32 (100%)	26 (100%)	25 (100%)
Lifetime hospitalizations Missing *n* = 4	No	38 (80.9%)	13 (76.5%)	25 (83.3%)	18 (75.0%)	20 (86.9%)
	Yes	9 (19.1%)	4 (23.5%)	5 (16.7%)	6 (25.0%)	3 (13.1%)
Number of hospitalizations	0.3 (±0.81)		0.5 (±1.1)	0.2 (±0.6)	0.4 (±0.9)	0.3 (±0.7)
Current pharmacological treatment	No	37 (74.0%)	11 (57.9%)	25 (78.1%)	18 (69.2%)	18 (72.0%)
	Yes	14 (26.0%)	8 (42.1%)	7 (21.9%)	8 (30.8%)	7 (28.0%)
Main current pharmacological treatment Missing *n* = 1	None	37 (74.0%)	12 (63.1%)	25 (80.7%)	18 (69.2%)	19 (79.0%)
	Sertraline	3 (6.0%)	1 (5.3%)	2 (6.5%)	2 (7.8%)	1 (4.2%)
	Escitalopram	2 (4.0%)	1 (5.3%)	1 (3.2%)	1 (3.8%)	1 (4.2%)
	Vortioxetine	1 (2.0%)	1 (5.3%)	0	1 (3.8%)	0
	Olanzapine	1 (2.0%)	0	1 (3.2%)	1 (3.8%)	0
	Benzodiazepines	1 (2.0%)	0	1 (3.2%)	1 (3.8%)	0
	Pregabalin	1 (2.0%)	1 (5.3%)	0	0	1 (4.2%)
	Fluoxetine	3 (6.0%)	2 (10.4%)	1 (3.2%)	2 (7.8%)	1 (4.2%)
	Melatonine	1 (2.0%)	1 (5.3%)	0	0	1 (4.2%)
Current poly-therapy Missing *n* = 1	No	40 (80.0%)	13 (68.4%)	27 (87.1%)	20 (76.9%)	20 (83.3%)
	Yes	10 (20.0%)	6 (31.6%)	4 (12.9%)	6 (23.1%)	4 (16.7%)
Current psychotherapy Missing *n* = 1	No	37 (74.0%)	13 (68.4%)	24 (77.4%)	19 (73.1%)	18 (75.0%)
	Yes	13 (26.0%)	6 (31.6%)	7 (22.6%)	7 (26.9%)	6 (25.0%)
Pharmacotherapy prescription	No	36 (70.6%)	9 (47.4%)	27 (84.4%)	19 (73.1%)	17 (68.0%)
	Yes	15 (29.4%)	10 (52.6%)	5 (15.6%)	7 (26.9%)	8 (32.0%)
Type of pharmacotherapy prescription Missing *n* = 2	None	36 (73.5%)	9 (47.4%)	27 (90.0%)	19 (73.2%)	17 (74.0%)
	Sertraline	3 (6.1%)	2 (10.5%)	1 (3.3%)	2 (7.7%)	1 (4.3%)
	Escitalopram	1 (2.0%)	1 (5.3%)	0	1 (3.8%)	0
	Vortioxetine	1 (2.0%)	1 (5.3%)	0	1 (3.8%)	0
	Zolpidem	5 (10.3%)	3 (15.7%)	2 (6.7%)	2 (7.7%)	3 (13.1%)
	Benzodiazepine	1 (2.0%)	1 (5.3%)	0	0	1 (4.3%)
	Pregabalin	2 (4.1%)	2 (10.5%)	0	1 (3.8%)	1 (4.3%)
Duration of the last pharmacological treatment (months)	1.2 (±2.57)		2.7 (±3.7)	0.4 (±1.0)	1.1 (±2.3)	1.4 (±2.9)
Medical comorbidity Missing *n* = 3	No	34 (70.8%)	15 (78.9%)	19 (65.5%)	19 (76.0%)	15 (65.2%)
	Yes	14 (29.2%)	4 (21.1%)	10 (34.5%)	6 (24.0%)	8 (34.8%)
Thyroid disorders Missing *n* = 3	No	47 (97.9%)	18 (94.7%)	29 (100%)	25 (100%)	22 (95.6%)
	Yes	1 (2.1%)	1 (5.3%)	0	0	1 (4.4%)
Hypercholesterolemia Missing *n* = 2	No	48 (98.0%)	17 (94.4%)	31 (100%)	25 (100%)	23 (95.8%)
	Yes	1 (2.0%)	1 (5.6%)	0	0	1 (4.2%)
Diabetes Missing *n* = 1	No	50 (100%)	19 (100%)	31 (100%)	25 (100%)	25 (100%)
Multiple medical comorbidities Missing *n* = 1	No	48 (96.0%)	19 (100%)	29 (93.5%)	24 (92.3%)	24 (100%)
	Yes	2 (4.0%)	0	2 (6.5%)	2 (7.7%)	0
Amenorrhea Missing *n* = 3	No	28 (58.3%)	14 (7.8%)	14 (46.7%)	10 (41.7%)	18 (75.0%)
	Yes	9 (18.8%)	1 (5.5%)	8 (26.6%)	8 (33.3%)	1 (4.2%)
	Estroprogestinic therapy	11 (22.9%)	3 (16.7%)	8 (26.6%)	6 (25.0%)	5 (20.8%)
Self-induced vomiting	No	36 (70.6%)	10 (52.6%)	26 (81.2%)	11 (44.0%)	25 (100%)
	Yes	15 (29.4%)	9 (47.4%)	6 (18.8%)	15 (66.0%)	0
Number of episodes of self-induced vomiting acts in the last month	3.7 (±11.28)		8.8 (±17.2)	0.6 (±2.7)	7.2 (±15.1)	0
Laxative misuse	No	42 (82.4%)	15 (78.9%)	27 (84.4%)	17 (65.4%)	25 (100%)
	Yes	9 (17.6%)	4 (21.1%)	5 (15.6%)	9 (34.6%)	0
Number of episodes of laxative use in the last month	1.4 (±4.91)		1.7 (±4.8)	1.2 (±5.0)	2.7 (±6.7)	0
Diuretic misuse	No	49 (96.1%)	18 (94.7%)	31 (96.9%)	24 (92.3%)	25 (100%)
	Yes	2 (3.9%)	1 (5.3%)	1 (3.1%)	2 (7.7%)	0
Number of episodes of diuretic use in the last month	1.1 (±5.59)		1.6 (±6.9)	0.9 (±4.9)	2.2 (±7.9)	0
Compulsive exercise	No	38 (74.5%)	14 (73.7%)	24 (75.0%)	13 (50.0%)	25 (100%)
	Yes	13 (25.5%)	5 (26.3%)	8 (25.0%)	13 (50.0%)	0
Number of episodes of compulsive exercises in the last month	5.3 (±10.48)		7.6 (±13.2)	4.0 (±8.5)	10.5 (±12.8)	0
Number of objective binge-eating episodes in the last month	7.4 (±15.0)		19.8 (±19.1)	0	5.9 (±12.0)	8.9 (±17.7)
Number of subjective binge-eating episodes in the last month	3.3 (±11.93)		6.1 (±18.1)	1.7 (±5.6)	2.5 (±6.6)	4.1 (±15.8)
Number of objective overeatings in the last month	1.0 (±3.82)		0.2 (±0.7)	1.4 (±4.7)	0.7 (±2.6)	1.2 (±4.8)
Dietary restraint (EDE subscale)	3.1 (±1.58)		2.9 (±1.5)	3.1 (±1.6)	3.2 (±1.5)	3.0 (±1.7)
Eating concern (EDE subscale)	2.9 (±1.33)		3.3 (±1.4)	2.7 (±1.3)	3.0 (±1.3)	2.8 (±1.4)
Body shape concern (EDE subscale)	3.9 (±1.53)		4.5 (±1.4)	3.5 (±1.5)	3.9 (±1.6)	3.9 (±1.5)
Weight concern (EDE subscale)	3.1 (±1.57)		3.8 (±1.3)	2.8 (±1.6)	3.0 (±1.6)	3.2 (±1.5)
EDE global score	3.2 (±1.17)		3.5 (±1.1)	3.1 (±1.2)	3.2 (±1.7)	3.3 (±1.2)
HAM-D	9.1 (±5.18)		10.5 (±4.5)	8.3 (±5.4)	8.2 (±5.2)	9.5 (±5.2)
HAM-A	8.8 (±5.39)		10.9 (±5.2)	7.6 (±5.2)	8.6 (±5.2)	9.0 (±5.6)
MADRS	12.5 (±6.41)		13.9 (±6.4)	11.7 (±6.4)	12.0 (±6.1)	13.0 (±6.8)
CGI-S	3.9 (±0.88)		3.9 (±0.9)	3.9 (±0.9)	4.1 (±0.6)	3.7 (±1.1)

Legend: AN = anorexia nervosa; BED = binge-eating disorder; BMI = body mass index; BN = bulimia nervosa; CGI-S = Clinical Global Impression-severity subscale; DUI = duration of untreated illness; ED = eating disorder; EDE = eating disorder examination; HAM-A = Hamilton Anxiety Rating Scale; HAM-D = Hamilton Depression Rating Scale; MADRS = Montgomery and Åsberg Depression Rating Scale; N = number; OCD = obsessive–compulsive disorder; OSFED = otherwise specified feeding and eating disorders; SUD = substance use disorder.

**Table 2:** Statistics of comparisons between groups defined by the presence/absence of objective binge-eating episodes or purging behaviors.

Variables		Objective Binge-Eating Episodes (Yes vs. No)			Purging Behaviors (Yes vs. No)		
		t or χ^2^	*p*	q-FDR	t or χ^2^	*p*	q-FDR
Age		0.095	**<0.001**	**0.004**	2.165	0.099	0.040
Gender	Male	1.893	0.169	0.043	0.397	0.529	0.061
	Female						
Education (years)		0.285	0.360	0.054	1.216	0.228	0.052
Work	Unemployed	1.328	0.580	0.074	7.072	0.029	0.016
	Student						
	Employed						
Marital Status Missing *n* = 4	Single/separated	0.038	0.846	0.093	0.201	0.654	0.072
	Married						
Smoke Missing *n* = 3	No	0.051	0.822	0.090	0.432	0.511	0.058
	Yes						
Number of cigarettes/day Missing *n* = 3		0.046	0.873	0.088	1.061	**0.030**	**0.032**
Dietary habits Missing *n* = 4	Omnivorous	6.574	0.087	0.032	2.820	0.420	0.051
	Vegetarian						
	Hyperproteic						
Age at onset		1.151	0.808	0.085	2.721	**0.015**	**0.024**
Diagnosis	AN	26.693	**<0.001**	**0.002**	10.186	0.017	0.015
	BN						
	BED						
	OSFED						
Duration of illness (months)		2.162	**0.019**	**0.031**	0.842	0.273	0.056
DUI (months)		0.823	0.114	0.038	1.672	**0.019**	**0.028**
BMI		4.422	0.245	0.046	2.173	**<0.001**	**0.004**
Family history of mental disorders Missing *n* = 2	No	0.993	0.319	0.061	0.214	0.644	0.070
	Yes						
Type of family history Missing *n* = 2	None	5.619	0.229	0.052	4.016	0.404	0.048
	Major depression						
	Anxiety disorders						
	SUD						
	EDs						
Obstetrical complications	None	3.715	0.294	0.058	4.003	0.261	0.032
	Low weight (<2500 g)						
	Premature (<32 weeks)						
	Cord hypoxia						
Lifetime substance use disorders Missing *n* = 1	No	1.389	0.239	0.053	1.745	0.187	0.028
	Yes						
Main type of substance use disorder Missing *n* = 1	None	6.597	0.159	0.040	4.950	0.292	0.037
	Cannabinoids						
	Opioids						
	Cocaine						
	Alcohol						
Poly-substance use disorders Missing *n* = 1	No	3.704	0.054	0.026	0.003	0.954	0.094
	Yes						
Psychiatric comorbidity Missing *n* = 2	No	5.628	0.018	0.012	0.783	0.376	0.045
	Yes						
Type of psychiatric comorbidity Missing *n* = 2	None	11.642	0.009	0.007	3.035	0.386	0.046
	Major depression						
	Anxiety disorders						
	OCD						
Multiple psychiatric comorbidity Missing *n* = 2	No	-	-	-	-	-	-
Lifetime psychotic symptoms Missing *n* = 1	No	-	-	-	-	-	-
Personality disorder Missing *n* = 6	No	6.016	0.014	0.010	0.621	0.431	0.053
	Yes						
Type of personality disorder Missing *n* = 6	None	11.042	0.026	0.018	5.108	0.276	0.035
	Schizotypic						
	Borderline						
	Hystrionic						
	Obsessive–compulsive						
Lifetime suicide attempts	No	-	-	-	-	-	-
Lifetime hospitalizations Missing *n* = 4	No	0.330	0.566	0.075	1.084	0.298	0.039
	Yes						
Number of hospitalizations		1.301	**0.012**	**0.023**	0.536	0.385	0.060
Current pharmacological treatment	No	2.350	0.125	0.037	0.047	0.828	0.084
	Yes						
Main current pharmacological treatment Missing *n* = 1	None	7.804	0.453	0.068	5.623	0.689	0.075
	Sertraline						
	Escitalopram						
	Vortioxetine						
	Olanzapine						
	Benzodiazepines						
	Pregabalin						
	Fluoxetine						
	Melatonine						
Current poly-therapy Missing *n* = 1	No	2.568	0.109	0.034	0.321	0.571	0.064
	Yes						
Current psychotherapy Missing *n* = 1	No	0.496	0.481	0.069	0.024	0.877	0.089
	Yes						
Pharmacotherapy prescription	No	7.864	**0.005**	**0.005**	0.158	0.691	0.076
	Yes						
Type of pharmacotherapy prescription Missing *n* = 2	None	12.704	0.048	0.024	3.474	0.747	0.080
	Sertraline						
	Escitalopram						
	Vortioxetine						
	Zolpidem						
	Benzodiazepine						
	Pregabalin						
Duration of the last pharmacological treatment (months)		3.189	**<0.001**	**0.011**	0.311	0.489	0.076
Medical comorbidity Missing *n* = 3	No	1.002	0.317	0.061	0.674	0.412	0.050
	Yes						
Thyroid disorders Missing *n* = 3	No	1.559	0.212	0.047	1.110	0.292	0.036
	Yes						
Hypercholesterolemia Missing *n* = 2	No	1.758	0.185	0.045	1.063	0.302	0.040
	Yes						
Diabetes Missing *n* = 1	No	-	-	-	-	-	-
Multiple medical comorbidities Missing *n* = 1	No	1.277	0.258	0.057	1.923	0.166	0.024
	Yes						
Amenorrhea Missing *n* = 3	No	5.032	0.081	0.030	7.821	0.020	0.015
	Yes						
	Estroprogestinic therapy						
Self-induced vomiting	No	4.703	0.030	0.019	20.433	**<0.001**	**0.003**
	Yes						
Number of episodes of self-induced vomiting acts in the last month		2.675	**<0.001**	**0.015**	2.380	**<0.001**	**0.012**
Laxative misuse	No	0.242	0.623	0.078	10.508	**0.001**	**0.009**
	Yes						
Number of episodes of laxative use in the last month		0.346	0.612	0.065	2.014	**<0.001**	**0.016**
Diuretic misuse	No	0.145	0.704	0.084	2.002	0.157	0.023
	Yes						
Number of episodes of diuretic use in the last month		0.424	0.398	0.058	1.414	**0.003**	**0.020**
Compulsive exercise	No	0.011	0.917	0.097	16.776	**<0.001**	**0.006**
	Yes						
Number of episodes of compulsive exercises in the last month		1.212	**0.004**	**0.019**	4.084	**<0.001**	**0.008**
Number of objective binge-eating episodes in the last month		5.895	**<0.001**	**0.008**	0.700	0.180	0.044
Number of subjective binge-eating episodes in the last month		1.296	**0.012**	**0.027**	0.470	0.216	0.048
Number of objective overeatings in the last month		1.162	**0.026**	**0.035**	0.435	0.395	0.068
Dietary restraint (EDE subscale)		0.400	0.777	0.081	0.443	0.558	0.080
Eating concern (EDE subscale)		1.360	0.651	0.069	0.475	0.453	0.072
Body shape concern (EDE subscale)		2.146	0.774	0.077	0.013	0.874	0.100
Weight concern (EDE subscale)		2.297	0.149	0.042	0.397	0.799	0.092
EDE global score		1.295	0.533	0.061	0.189	0.662	0.084
HAM-D		1.449	0.302	0.050	0.502	0.870	0.096
HAM-A		2.122	0.966	0.100	0.201	0.754	0.088
MADRS		1.125	0.754	0.073	0.491	0.385	0.064
CGI-S		0.261	0.923	0.096	1.531	0.037	0.036

Legend: AN = anorexia nervosa; BED = binge-eating disorder; BMI = body mass index; BN = bulimia nervosa; CGI-S = Clinical Global Impression-severity subscale; χ^2^ = chi-square; DUI = duration of untreated illness; ED = eating disorder; EDE = Eating Disorder Examination; HAM-A = Hamilton Anxiety Rating Scale; HAM-D = Hamilton Depression Rating Scale; MADRS = Montgomery and Åsberg Depression Rating Scale; OCD = obsessive–compulsive disorder; OSFED = otherwise specified feeding and eating disorders; *p* = *p*-value; SUD = substance use disorder; t = Student’s *t*-test. In bold, statistically significant *p*-value (≤0.05). For the total, sample mean and ±standard deviations (into bracket) are reported for continuous variables and frequencies with percentages (into brackets) for qualitative variables. We have reported in bold statistically significant *p* and q-FDR values resulting from multiple comparison methods based on Benjamini–Hochberg False Discovery Rate.

**Table 3:** Binary logistic regression model on factors associated with the presence of purging behaviors.

Variables	B	S.E.	Wald	*p*	OR	95% CI for OR
Number of cigarettes/day	0.062	0.078	0.629	0.428	1.064	0.913–1.240
Age at onset	0.143	0.076	3.522	0.061	1.153	0.994–1.339
DUI (months)	0.021	0.016	1.792	0.181	1.021	0.990–1.053
BMI	−0.127	0.060	4.467	**0.035**	0.881	0.783–0.991

Legend: Presence versus absence of purging episodes was considered as the dependent variable. B = regression coefficient; CI = confidence interval; DUI = duration of untreated illness; OR = odds ratio; S.E. = standard error of B; Wald = Wald statistics. In bold, statistically significant *p*-value (≤0.05).

**Table 4:** Binary logistic regression model on factors associated with the presence of objective binge-eating episodes.

Variables	B	S.E.	Wald	*p*	OR	95% CI for OR
Age	0.008	0.072	0.014	0.907	1.009	0.875–1.162
Duration of illness	0.005	0.009	0.330	0.566	1.005	0.988–1.022
Duration of last pharmacological treatment (months)	0.450	0.225	3.996	**0.046**	1.569	1.009–2.440
Number of hospitalizations	0.256	0.453	0.321	0.571	1.292	0.532–3.140

Legend: Presence versus absence of purging episodes was considered as the dependent variable. B = regression coefficient; CI = confidence interval; OR = odds ratio; S.E. = standard error of B; Wald = Wald statistics. In bold, statistically significant *p*-value (≤0.05).

## Data Availability

The raw data supporting the conclusions of this article will be made available by the authors upon request.
